# Intraluminal chloride regulates lung branching morphogenesis: involvement of PIEZO1/PIEZO2

**DOI:** 10.1186/s12931-023-02328-2

**Published:** 2023-02-05

**Authors:** Ana N. Gonçalves, Rute S. Moura, Jorge Correia-Pinto, Cristina Nogueira-Silva

**Affiliations:** 1 School of Medicine, Life and Health Sciences Research Institute (ICVS), University of Minho, Campus de Gualtar, Gualtar, 4710-057 Braga, Portugal; 2 Life and Health Sciences Research Institute/3B’s-PT Government Associate Laboratory, Braga/Guimarães, Portugal; 3 Department of Pediatric Surgery, Hospital de Braga, Braga, Portugal; 4 Department of Obstetrics and Gynecology, Hospital de Braga, Braga, Portugal

**Keywords:** Chloride, Branching, Lung development, Lung fluid, Mechanotransduction

## Abstract

**Background:**

Clinical and experimental evidence shows lung fluid volume as a modulator of fetal lung growth with important value in treating fetal lung hypoplasia. Thus, understanding the mechanisms underlying these morphological dynamics has been the topic of multiple investigations with, however, limited results, partially due to the difficulty of capturing or recapitulating these movements in the lab. In this sense, this study aims to establish an ex vivo model allowing the study of lung fluid function in branching morphogenesis and identify the subsequent molecular/ cellular mechanisms.

**Methods:**

Ex vivo lung explant culture was selected as a model to study branching morphogenesis, and intraluminal injections were performed to change the composition of lung fluid. Distinct chloride (Cl^−^) concentrations (5.8, 29, 143, and 715 mM) or Cl^−^ channels inhibitors [antracene-9-carboxylic acid (A9C), cystic fibrosis transmembrane conductance regulator inhibitor172 (CFTRinh), and calcium-dependent Cl^−^ channel inhibitorA01 (CaCCinh)] were injected into lung lumen at two timepoints, day0 (D0) and D2. At D4, morphological and molecular analyses were performed in terms of branching morphogenesis, spatial distribution (immunofluorescence), and protein quantification (western blot) of mechanoreceptors (PIEZO1 and PIEZO2), neuroendocrine (bombesin, ghrelin, and PGP9.5) and smooth muscle [alpha-smooth muscle actin (α-SMA) and myosin light chain 2 (MLC2)] markers.

**Results:**

For the first time, we described effective intraluminal injections at D0 and D2 and demonstrated intraluminal movements at D4 in ex vivo lung explant cultures. Through immunofluorescence assay in in vivo and ex vivo branching morphogenesis, we show that PGP9.5 colocalizes with PIEZO1 and PIEZO2 receptors. Fetal lung growth is increased at higher [Cl^−^], 715 mM Cl^−^, through the overexpression of PIEZO1, PIEZO2, ghrelin, bombesin, MLC2, and α-SMA. In contrast, intraluminal injection of CFTRinh or CaCCinh decreases fetal lung growth and the expression of PIEZO1, PIEZO2, ghrelin, bombesin, MLC2, and α-SMA. Finally, the inhibition of PIEZO1/PIEZO2 by GsMTx4 decreases branching morphogenesis and ghrelin, bombesin, MLC2, and α-SMA expression in an intraluminal injection-independent manner.

**Conclusions:**

Our results identify PIEZO1/PIEZO2 expressed in neuroendocrine cells as a regulator of fetal lung growth induced by lung fluid.

**Supplementary Information:**

The online version contains supplementary material available at 10.1186/s12931-023-02328-2.

## Background

Physical forces exerted on the developing fetal lung, namely by intraluminal lung fluid and peristaltic airway contractions, are important regulators of fetal lung branching morphogenesis. Lung fluid and its in utero intraluminal hydraulic pressure have two sources: amniotic fluid and secretions of the epithelial cells into the airway lumen, which are osmotically driven by active chloride (Cl^−^) secretion through Cl^−^ channels; this gives rise to a continuous forward flow of lung liquid that drains into the amniotic fluid. The physiological circulation of lung fluid filling the air spaces is critical to lung development. In fact, if it is disturbed, lung growth and maturation are impaired. For instance, excess fluid drainage during fetal life or a decrease of fluid pressure due to premature rupture of the membranes or oligohydramnios are associated with lung hypoplasia with underbranched lungs, which is a major cause of respiratory insufficiency and mortality in newborns [1–8]. In opposition, experimental evidence shows the increase of lung fluid volume as a promotor of fetal lung growth [2, 9]. In fact, prenatal tracheal occlusion increases lung fluid volume, luminal pressure, and expansion and, consequently, enhances the branching rate [5, 10–13]. This evidence allowed the development of fetoscopic endoluminal tracheal occlusion (FETO) as a treatment for the more severe cases of pulmonary hypoplasia in the context of congenital diaphragmatic hernia (CDH) [13–16].

Molecular studies have been performed to determine the mechanisms underlying lung fluid production and pulmonary expansion. In brief, the lung fluid is produced by a mechanism dependent on sodium–potassium adenosine triphosphatase (Na^+^/K^+^-ATPase) pumps and Na^+^/K^+^/2chloride (Cl^−^) co-transporters located on the basolateral surface of pulmonary epithelial cells [17–21], that stimulate the apical Cl^−^ secretion via cystic fibrosis transmembrane conductance regulator (CFTR) or calcium-dependent chloride channel (CaCC). Finally, it is the increase of intraluminal Cl^−^ concentration ([Cl^−^]) that favors the movement of sodium and water into the lumen and promotes lung liquid formation and the consequent pulmonary expansion [5, 18–25]. In addition, inhibition of apical ionic channels, such as CFTR, transmembrane protein 16A (TMEM16A), chloride channel2 (ClC2), or the extracellular calcium receptor (CaR), induces key morphological defects in branching morphogenesis [18, 20, 26–30].

Recently, an emergent area, mechanotransduction, showed that cells are able to translate a mechanical stimulus, like pressure, into biochemical signaling. However, the mechanisms by which pressure is sensed in the lung have not been determined yet. In the fetal lung, smooth muscle cells are essential for peristaltic airway contractions, while the pulmonary neuroendocrine cells (PNECs)/ neuroepithelial bodies (NEBs) are indicated as chemo- and mechano-sensors, particularly during the perinatal period. Indeed, the peristaltic airway contractions generate not only the flow of intraluminal fluid but also the periodic distension and relaxation of the end buds essential for branching morphogenesis [31–34]. Oppositely, PNECs/NEBs are promotors of in vivo and ex vivo fetal lung growth [35–39] and sensors for hypoxia, hypercapnia, acidosis, or airway stretch [40] with undefined functions in fetal lung development. A recent publication showed that PIEZO2, a known mechanosensor [41–43], is expressed in NEBs, indicating that NEBs can sense mechanical stretch [41]. This study also reports the presence of PIEZO2 in sensory neurons and its importance in regulating lung expansion and efficient neonatal respiration in a mechanism dependent on the central nervous system [41]. However, the inactivation of PIEZO2 in sensory neurons, but not in PNECs/NEBs, was essential for respiratory transition at birth [41], maintaining the importance of stretch sensation by PNECs unclear.

PIEZO proteins, PIEZO1 and PIEZO2, are mechanically activated cation channels that form homomultimeric complexes sufficient to mediate mechanically induced currents [44–46]. Previous work showed PIEZO1 as essential in the regulation of basal blood pressure and normal cellular volume in red blood cells in adulthood [43, 47], whereas PIEZO2 mediated the sensory processes [48–50] and the respiratory physiology [41].

In this context, to investigate the mechanotransduction signaling intrinsic to fetal lung growth, we explore the neuroendocrine cells and the mechanoreceptors as mediators of intraluminal fluid composition during branching morphogenesis.

## Methods

### Animals

Female Sprague–Dawley rats (225 g; Charles-River; Spain) were maintained in appropriate cages under controlled conditions and fed with commercial solid food. The rats were mated and checked daily for vaginal plug. The day of plugging was defined as embryonic day (E) 0.5 for time dating purposes. Embryos were dissected at E13.5 or E17.5, and the embryonic lungs were removed for further analysis.

### Lung explant cultures

Harvesting and dissection of E13.5 lungs were made in PBS under a dissection microscope (Leica MZFLIII, Switzerland). Lungs were then transferred to the nucleopore membranes (Cat No. TETP01300, Whatman, USA) and cultured in a complete medium [50% DMEM low glucose, 50% nutrient mixture F-12 (Gibco, USA) supplemented with 100 µg/mL glutamine (Cat. No. 25030081, Gibco, USA), 100 units/mL penicillin–streptomycin, (Cat. No. 15140122, Gibco, USA), 0.25 mg/mL l-ascorbic acid (Cat No. A4403, Sigma-Aldrich, USA) and 10% fetal bovine serum (FBS) (Cat No. 26140079, Gibco, USA)] [51]. The fetal lung explants were incubated in a 5% CO_2_ incubator at 37 °C for 96 h, and the medium was replaced every 48 h. Explants were processed for immunofluorescence or western blot assay.

### Lung fluid manipulation

#### Distinct chloride concentration

According to in vivo [23] and ex vivo [20, 52, 53] studies, 143 mM Cl^−^ was defined as basal [Cl^−^]. The intraluminal [Cl^−^] manipulation was achieved using three experimental concentrations: 5.8, 29, and 715 mM Cl^−^. Lungs were randomly assigned to one of four experimental groups (n ≥ 12 per condition). After intraluminal injections at day 0 (D0) and D2, morphological and molecular dynamics were determined at D4.

To strictly manipulate the [Cl^−^] maintaining a similar concentration of the remaining ions, the following chemical compounds were used: potassium chloride (KCl, Cat No. 7447-40-7, Merck, Germany), magnesium chloride (MgCl_2_, Cat No. 7786-30-3, Merck, Germany), calcium chloride (CaCl_2_, Cat No. C1016-100G, Sigma-Aldrich, USA), potassium d-Gluconate (Cat No. G8270-100G, Sigma-Aldrich, USA), MgSO_4_ (Cat No. M7506 Sigma-Aldrich, USA) and calcium d-gluconate (Cat No. C8231-100G, Sigma-Aldrich, USA). Specifically, KCl, MgCl_2_, and CaCl_2_ were used as donors of Cl^−^, K^+^, Mg^2+^, and Ca^2+^, while potassium d-gluconate, MgSO_4_, and calcium d-gluconate worked as replace compounds as demonstrated in the Additional file 1: Table S1a, b. Thus, the relative influence of the different chemical compounds for the lower and higher [Cl^−^] were as follow (in mM, adapted from [54]): 5.8 mM Cl^−^: KCl (5.711), MgCl_2_ (0.049), CaCl_2_ (0.041), d-glucose (10.000, Cat No. G8270-1KG, Sigma-Aldrich, USA), HEPES (5.000, Cat No. H3375-25G, Sigma-Aldrich, USA), potassium d-gluconate (698.290), MgSO_4_ (5.950, Cat No. M7506-1KG-M, Sigma-Aldrich, USA), Calcium d-gluconate (4.960, Cat No. C8231-100G, Sigma-Aldrich, USA); 715 mM Cl^−^: KCl (704.000), MgCl_2_ (6.000), CaCl_2_ (5.000), d-glucose (10.000), HEPES (5.000) as shown in Additional file 1: Table S1a, b.

#### Chloride channel inhibitors

Anthracene-9-carboxylic acid (A9C, Cat No. A89405, Sigma-Aldrich, USA) (Valenzuela et al., 2000; Al Khamici et al., 2015), CFTRinh (Cat No. C2992, Sigma-Aldrich, USA) (Li et al., 2007; Melis et al., 2014) or calcium-dependent Cl^−^ channel inhibitor A01 (CaCCinh, Cat No. SML0916, Sigma-Aldrich, USA) (Boedtkjer et al., 2015; Nakazawa et al., 2016) were dissolved in dimethyl sulfoxide (DMSO, Cat No. D8418, Sigma-Aldrich, USA) according to the manufacture’s protocol guidelines. Lung explants were randomly assigned to one of four experimental groups: A9C (10 µM), CFTRinh (5 µM), CaCCinh (10 µM), or matching volumes of DMSO for control (n ≥ 12 per condition).

Inhibitors and DMSO were diluted in a standard solution containing (in mM): sodium chloride (135.000, NaCl, Cat No. 7647-14-5, Merck, Germany), KCl (5.000), MgCl_2_ (1.200), CaCl_2_ (1.000), d-glucose (10.000), HEPES (5.000) were used as vehicle according to the previously published work adapted from [20].

### Intraluminal injections

Pulmonary tissues were punctured for intraluminal injections. For that, borosilicate glass capillaries (1.55 mm outer diameter, 1.15 mm inner diameter; HIRS9201590, VWR International, USA) were pulled using Flaming Brown Micropipette Puller (P500, Heat 545, Vel 13, Del 10; Model P-97, Sutter Instrument Co., USA). Lung explants were randomly selected and, under the stereoscopic dissecting microscope (Olympus SZX16 stereomicroscope), the intraluminal injections were performed on day0 (D0) and day2 (D2). The pulled borosilicate glass capillary was filled with one of the experimental solutions marked with trypan blue (Cat No. T8154, Sigma-Aldrich, USA), and the capillary was then slowly inserted into the lumen. The presence of trypan blue in the lumen was indicative of a successful procedure. Only the lung explants with effective injections at D0 and D2 and perfectly placed in the nucleopore membrane were considered for analysis.

### PIEZO1/PIEZO2 inhibition

GsMTx4 (Cat No. ab141871, Abcam, UK), a selective PIEZO1/PIEZO2 inhibitor, was diluted in water. At the final concentration of 5 µM, GsMTx4 was added to the culture medium on the day of intraluminal injections, D0 and D2, in accordance with the previously published work [42, 55, 56]. Lungs were randomly assigned to one of four experimental groups (n ≥ 12 per condition).

### Morphometric analysis

The branching morphogenesis was monitored daily by photographing the explants. At D0 (0 h) and D4 (96 h) of culture, the branching of all lung explants was determined by counting the number of peripheral airway epithelial buds of the developing respiratory tree [57]. For the morphometric analysis, the internal perimeter of the lung (epithelium) was assessed at D0 and D4 using Axion-Vision Rel 4.3 (Carl Zeiss GmbH, Germany).

### Immunofluorescence

For immunostaining, lungs at E17.5 and from the lung explant cultures at D4 were fixed in 4% paraformaldehyde for 2 h or 15 min, respectively. Whole lungs and explants were then embedded in OCT (OCT compound, Cat No. 4582, Scigen, UK), sectioned (4 μm), and placed on SuperFrost^®^Ultra Plus slides (11976299, Thermo Scientific, UK).

Double immunostained using a 3-day protocol was performed (adapted from [58, 59]). Slides were first boiled in 10 mM citrate buffer (Cat No. AP-9003-125, Thermo Scientific, UK) for 20 min (in vivo samples) or 5 min (explant). Samples were blocked by incubation in 20% bovine serum albumin (Cat No. A3294, Sigma-Aldrich, USA) and 0.5% Triton X-100 (Cat No. 9036-19-5, Merck, Germany) for 4 h, followed by 36 h of incubation with primary antibodies at room temperature (RT). Sections were then washed and incubated with the corresponding secondary antibodies for 12 h in 1% BSA in PBS at RT. Finally, samples were washed in PBS1x, incubated with 4′,6-diamidino-2-phenylindole (DAPI, Cat No. D1306, Life Technologies, USA) for 1 min at RT, and mounted in PermaFluor™ Aqueous Mounting Medium (Cat No. TA-006-FM, Life Technologies, USA). Visualization and image capture of immunofluorescence staining was performed using an Olympus Widefield Upright Microscope BX61 (Olympus Corporation, Japan).

The primary antibodies used were PGP9.5 (1:150, Cat No. ab72911, Abcam, UK), PIEZO1 (1:50, Cat No. NBP1-78537, Novus Biologicals, USA), and PIEZO2 (1:50, Cat No. NBP1-78624, Novus Biologicals, USA). Negative control reactions included the omission of the primary antibody. The secondary antibodies were: Alexa Fluor 647-conjugated donkey anti-rabbit IgG(H + L) (1:500, Cat No. A31573, Life Technologies, USA) and Alexa Fluor plus 488-conjugated goat anti-mouse IgG(H + L) (1:500, Cat No. A32723, Life Technologies, USA). Different and unrepeated in vivo samples or lung explants were randomly selected, n ≥ 4 per stage or condition/antibody.

### Western blot

Lungs explants were processed for western blot analysis according to the previously described methods [51]. In brief, 15 µg of protein were loaded onto 10% acrylamide mini gels, electrophoresed at 100 V at room temperature, and then transferred to nitrocellulose membranes (HybondTM-C Extra, GE Healthcare Life Sciences, UK). Blots were blocked in 5% bovine serum albumin and probed with primary antibodies to ghrelin (1:250, ON, 4 ºC; Cat No. sc10368, Santa Cruz Biotechnology Inc., USA), bombesin (1:250, ON, 4 ºC; Cat No. H00002922-MO3, Novus Biologicals, USA), PIEZO1 (1:250, ON, 4 ºC; Cat No. HPA047185, Sigma-Aldrich, USA), PIEZO2 (1:250, ON, 4 ºC; Cat No. HPA040616, Sigma-Aldrich, USA), myosin light chain 2 (MLC2, 1∶250; ON, 4 ºC; Cat No. #3672, Cell Signaling Technology Inc., USA), and alpha-smooth muscle actin (α-SMA, 1:500, ON, 4 ºC; Cat No. NBP2-33006, Novus Biologicals, USA) according to the manufacturer's instructions. For loading control, blots were probed with GAPDH (1∶5000; Cat No. MAB5718, R&D system, USA). After this, membranes were incubated with the corresponding secondary antibodies, developed with Clarity West ECL substrate (Cat No. 1705060, Bio-Rad, USA), and the chemiluminescent signal was captured by the Chemidoc XRS (Bio-Rad, USA) [51].

Quantitative analysis was performed with Quantity One 4.6.5 1-D Analysis Software (Bio-Rad, USA). Three independent experiments were performed (n ≥ 4 were used per antibody/condition).

### Statistical analysis

All quantitative data are presented as mean ± standard deviation (SD). One-way ANOVA was performed for the number of peripheral airway buds, epithelial perimeter, and protein expression levels on [Cl^−^] (5.8, 29, 143, 715 mM), and Cl^−^ channels inhibitors (SS, A9C, CFTRinh, CaCCinh). Two-way ANOVA was used in the analysis of both morphological (number of peripheral airway buds, epithelial perimeter) and the molecular (protein expression levels) effect after GsMTx4 exposure. The parametric test assumptions were previously verified, and an additional LSD test was used for post-test analysis. Statistical analysis was performed using the statistical software IBM SPSS Statistics 26.0. Statistical significance was confirmed at p < µ0.05, γ0.01, β0.001, and α0.0001.

## Results

### Intraluminal injection in ex vivo lung explant cultures

To establish the intraluminal injections in ex vivo lung explant cultures, lung tissue was punctured using pulled borosilicate glass capillaries, and, for the first time, effective intraluminal injections at D0 and D2 were performed, as demonstrated in the Additional file 1: Movie S1, and Additional file 2: Movie S2, respectively. Furthermore, dynamic luminal movements were observed at D4 (an additional movie file shows this in more detail, see Additional file 3: Movie S3) that recognized the existence of fetal lung liquid in ex vivo lung explant cultures.

### Luminal chloride as a modulator of branching morphogenesis

To study the role of intraluminal composition in branching morphogenesis, ex vivo lung explants were cultured for 4 days after injection of distinct [Cl^−^]: 5.8, 29, 143, 715 mM Cl^−^ or Cl^−^ channels inhibitors: SS, A9C, CFTRinh, CaCCinh.

**Fig. 1:**
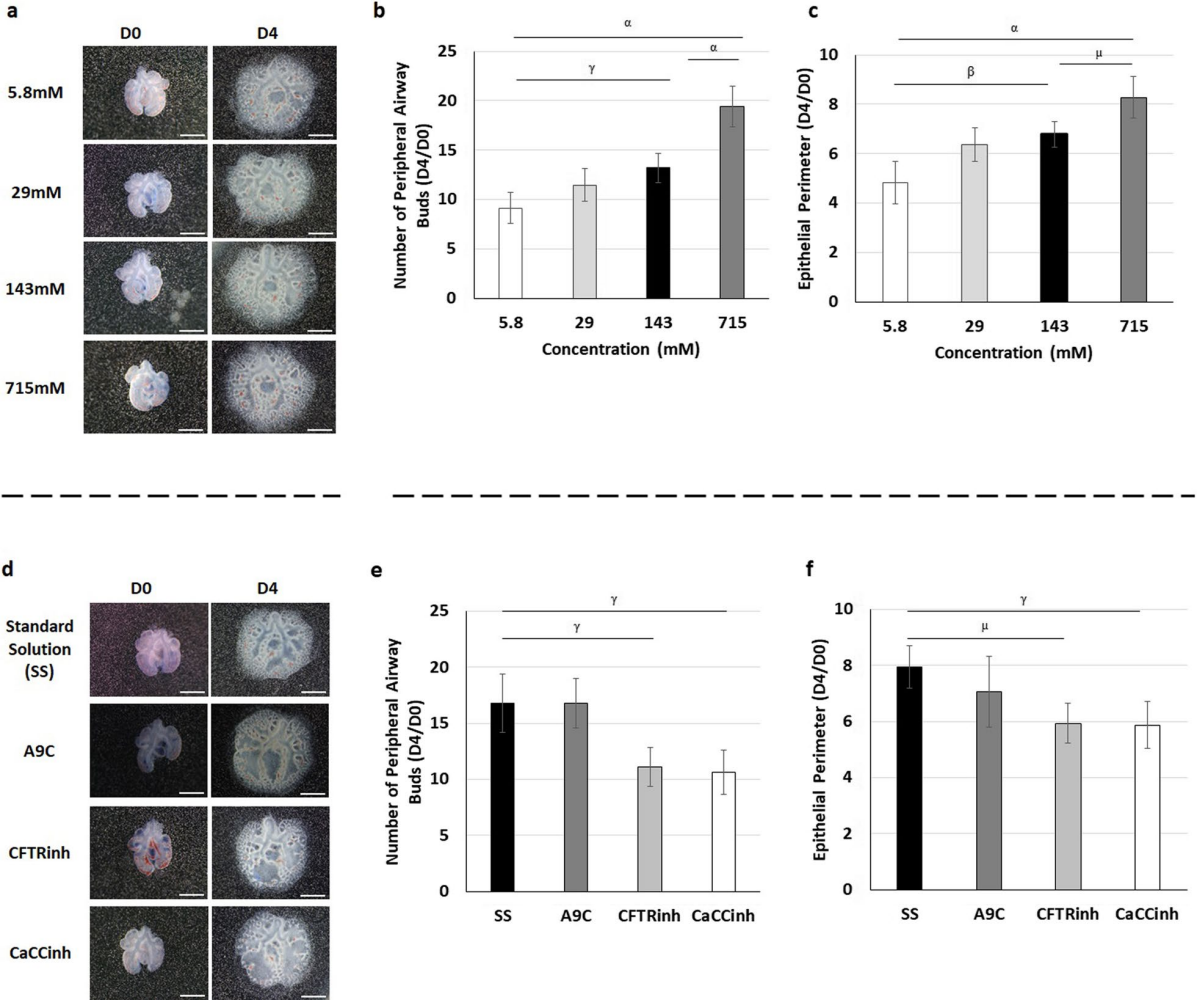
Intraluminal chloride modulates branching morphogenesis. Upper panel: **a** represents lung explants on day0 (D0) and day4 (D4) after intraluminal injection of distinct chloride concentrations ([Cl^−^]): 5.8, 29, 143, and 715 mM Cl^−^. **b**, **c** Morphometric analysis of **b** number of peripheral airway buds and **c** epithelial perimeter at the different [Cl^−^]. Lower panel: **d** represents the fetal lung explants after intraluminal injection of distinct Cl^−^ channels inhibitors: anthracene-9-carboxylic acid (A9C) to transmembrane protein 16A TMEM16A; cystic fibrosis transmembrane conductance regulator inhibitor172 (CFTRinh) to CFTR; and calcium-dependent Cl^−^ channel inhibitorA01 (CaCCinh) to CaCC. **e**, **f** Morphometric analysis of **e** number of peripheral airway buds and **f** epithelial perimeter. Lungs were randomly assigned to one of eight experimental groups (n ≥ 12 per condition). Results are expressed as the ratio of D4 and D0 (D4/D0) and presented as mean ± SD. Black rectangles define the control group. Scale bar, 1 mm. p < ^α^0.0001, ^β^0.001, ^γ^0.01, ^µ^0.05

Morphometric analysis revealed an opposite effect in branching morphogenesis after injection of 5.8 and 715 mM Cl^−^, indicated by the ratio of D4 and D0 in the number of peripheral airway buds (Fig. 1a, b) and epithelial perimeter (Fig. 1c). Specifically, when compared to basal [Cl^−^], 5.8 mM inhibits, whereas 715 mM Cl^−^ stimulates both the number of peripheral airway buds (Fig. 1b) and the epithelial perimeter (Fig. 1c). Unexpectedly, 29 mM injection did not change branching morphogenesis at D4 compared to basal, 143 mM Cl^−^ (Fig. 1a–c).

Regarding the Cl^−^ channels inhibitors, an important inhibitory effect in terms of the number of peripheral airway buds (Fig. 1d–e) and epithelial perimeter (Fig. 1f) was visualized after CFTRinh or CaCCinh injections when compared with SS, while unchanged lung growth was observed after A9C luminal injection (Fig. 1d–f).

### PIEZO1 and PIEZO2 are expressed in pulmonary neuroendocrine cells during branching morphogenesis

**Fig. 2:**
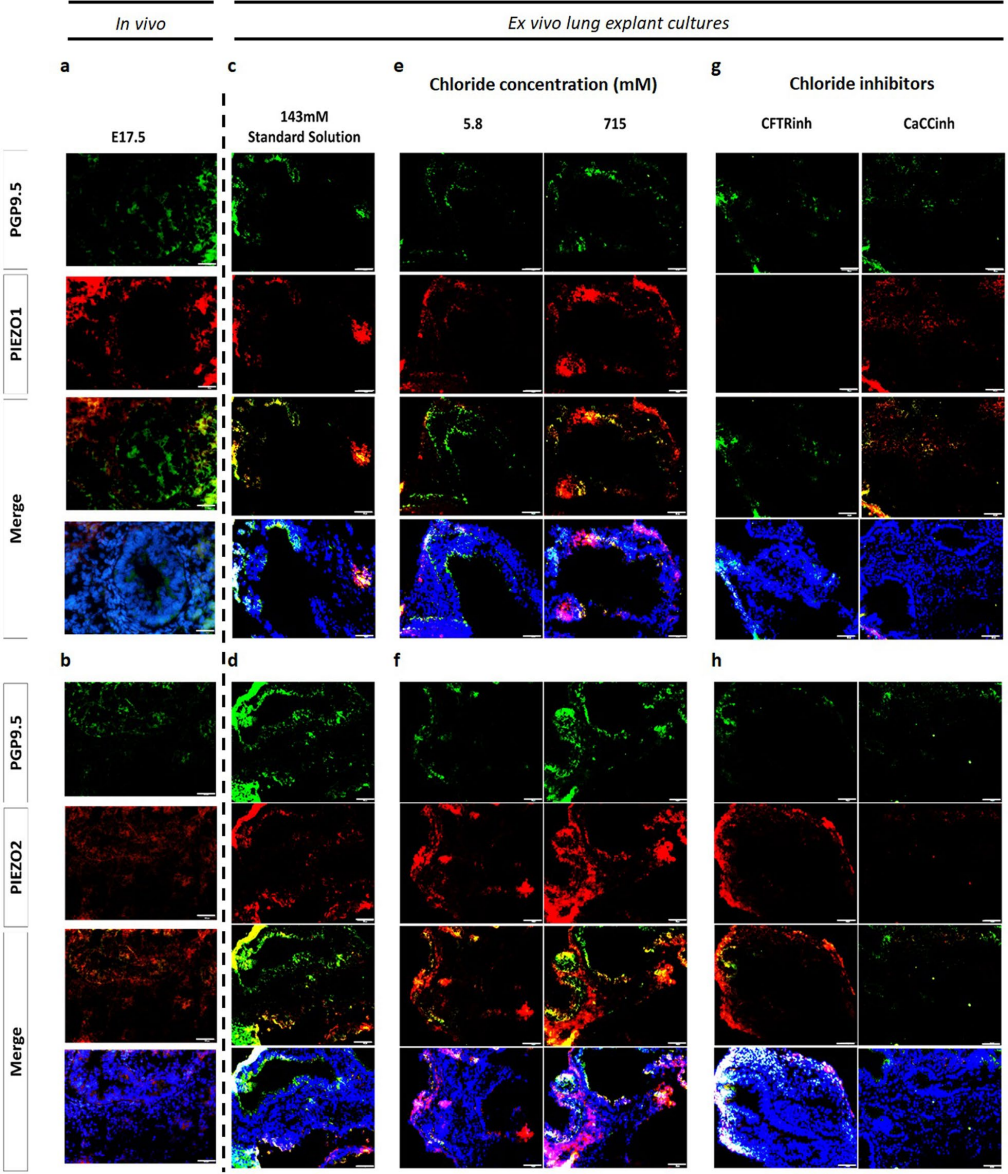
Spatial distribution of PIEZO1 and PIEZO2 in in vivo and ex vivo branching morphogenesis. Representative examples of immunofluorescence assay for PIEZO1, PIEZO2, and PGP9.5 staining at **a**, **b** embryonic day (E)17.5; and after intraluminal injections of **c**, **d** 143 mM Cl^−^ or standard solution (SS); **e**, **f** different chloride concentrations ([Cl^−^]): 5.8 and 715 mM Cl^−^; and **g**, **h** Cl^−^ channel inhibitors: cystic fibrosis transmembrane conductance regulator inhibitor172 (CFTRinh) to CFTR; and calcium-dependent Cl^−^ channel inhibitor A01 (CaCCinh) to CaCCs. 143 mM Cl^−^ and standard solution (SS) represent the control condition for [Cl^−^] and Cl^−^ channels inhibitors, respectively, in which similar spatiotemporal distribution was observed. n ≥ 4 per stage or condition/antibody for whole lungs and lung explants. Scale bar 50 µm

To explore the molecular mechanism under branching morphogenesis and intraluminal composition, the spatial distribution of mechanosensors (PIEZO1 and PIEZO2) and neuroendocrine cell marker (PGP9.5) were determined in both in vivo and ex vivo branching morphogenesis. Immunofluorescence assay disclosed the colocalization of PIEZO1 (Fig. 2a) and PIEZO2 (Fig. 2b) with PGP9.5 at E17.5 and after intraluminal injection of SS or 143 mM Cl^−^ in ex vivo lung explant cultures (Fig. 2c-d). Concerning the [Cl^−^] and Cl^−^ channels inhibitors, the similar expression profile observed for PIEZO1 (Fig. 2e) and PIEZO2 (Fig. 2f) in PGP9.5 + cells at 5.8 and 715 mM Cl^−^ contrast with the more restricted PIEZO1 (Fig. 2g) and PIEZO2 (Fig. 2h) pattern visualized in neuroendocrine cells after injection of CFTRinh or CaCCinh.

### Intraluminal chloride concentration regulates ghrelin, bombesin, PIEZO1, and PIEZO2, expression levels

Since the PIEZO1 and PIEZO2 are expressed in neuroendocrine cells in branching morphogenesis, we then quantified by western blot the relative expression levels of receptors: PIEZO1, PIEZO2; and neuroendocrine products: ghrelin and bombesin, at the above-mentioned experimental conditions.

**Fig. 3:**
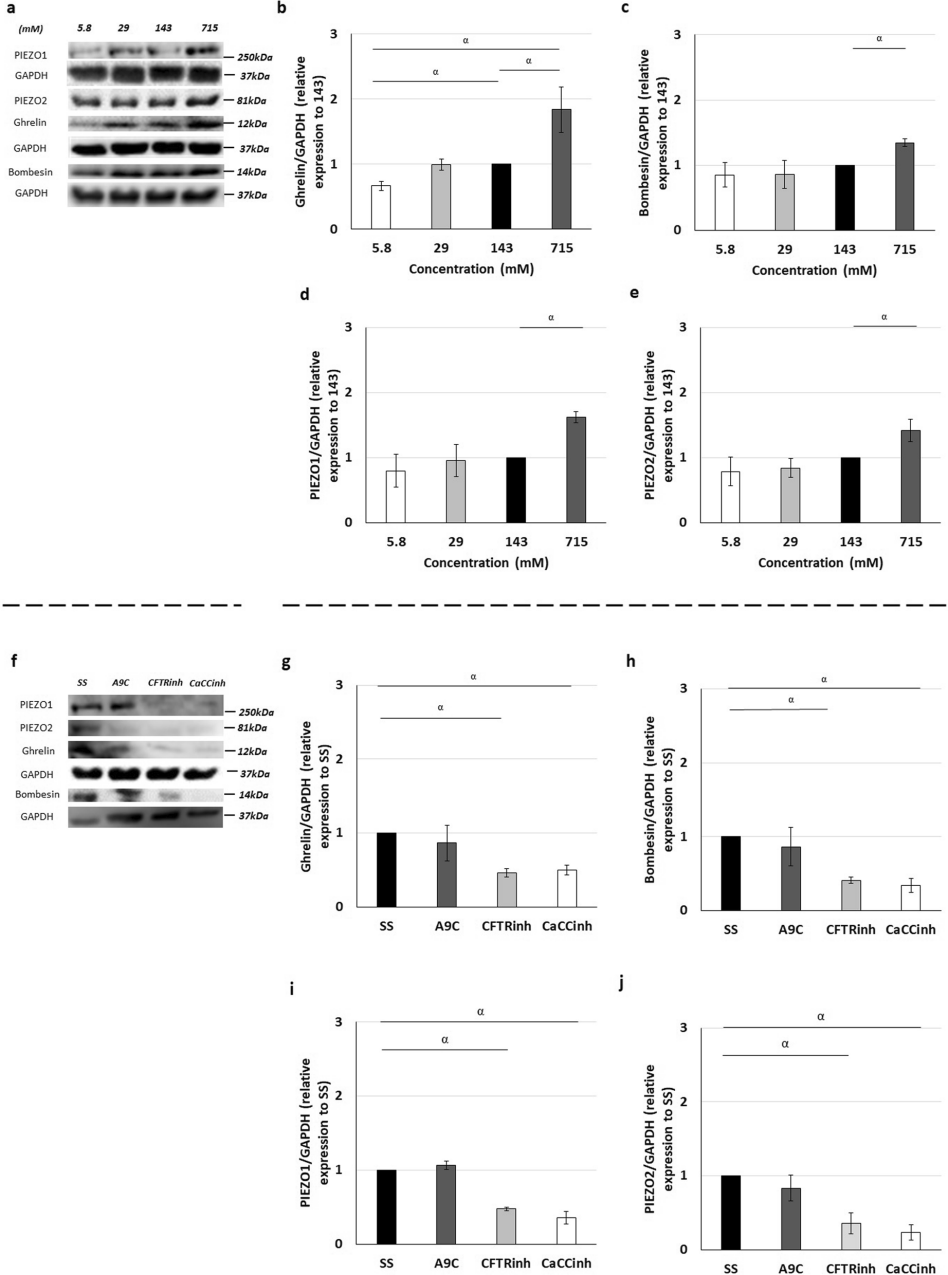
Intraluminal chloride modulates the expression of ghrelin, bombesin, PIEZO1, and PIEZO2. **a**–**e** Upper panel represents the main effects of the distinct chloride concentrations ([Cl^−^]). **a** Examples of representative blots are shown. **b**–**e** Relative expression levels of **b** ghrelin, **c** bombesin, **d** PIEZO1, and **e** PIEZO2. **f**–**j** Lower panel shows the molecular effect of intraluminal Cl^−^ channels inhibition by anthracene-9-carboxylic acid (A9C) to transmembrane protein 16A (TMEM16A); cystic fibrosis transmembrane conductance regulator inhibitor172 (CFTRinh) to CFTR and calcium-dependent Cl^−^ channel inhibitor A01 (CaCCinh) to CaCCs. **f** Examples of representative blots are shown. **g**–**j** Protein expression levels for **g** ghrelin, **h** bombesin, **i** PIEZO1 and **j** PIEZO2. Black rectangles define the control group. Each lane represents a pooled-tissue sample, and relative expression levels were determined against GAPDH. n ≥ 4 were used per antibody/condition. Results are presented as mean ± SD. Symbols indicate the main effects and non-redundant interactions of the one-way ANOVA. p < ^α^0.0001

In comparison with 143 mM Cl^−^, 715 mM was an inductor of ghrelin (Fig. 3a, b), bombesin (Fig. 3c), PIEZO1 (Fig. 3d), and PIEZO2 (Fig. 3e) expression, whereas 5.8 mM Cl^−^ inhibited the relative expression levels of ghrelin (Fig. 3a–e). Unchanged molecular profiles in terms of ghrelin (Fig. 3b), bombesin (Fig. 3c), PIEZO1 (Fig. 3d), or PIEZO2 (Fig. 3e) were detected analyzing 29 mM versus 143 mM Cl^−^.

Regarding the Cl^−^ channels inhibition, distinct effects on ghrelin (Fig. 3f, g), bombesin (Fig. 3h), PIEZO1 (Fig. 3i), and PIEZO2 (Fig. 3j) expression were visualized after inhibition of TMEM16A by A9C, CFTR by CFTRinh, and CaCC by CaCCinh as demonstrated in Fig. 3f–j. Specifically, compared with SS, the injection of CFTRinh or CaCCinh were inhibitors of ghrelin (Fig. 3f, g), bombesin (Fig. 3h), PIEZO1 (Fig. 3i), and PIEZO2 (Fig. 3j) expression, whereas no significant modifications in the protein expression levels were observed after A9C injection (Fig. 3f–j).

### PIEZO1 and PIEZO2 control branching morphogenesis

To evaluate the functional role of PIEZO1 and PIEZO2 in branching morphogenesis, the culture medium was supplemented with GsMTX4, a known pharmacological inhibitor of PIEZO1 and PIEZO2, on the day of intraluminal injections, D0 and D2.

**Fig. 4:**
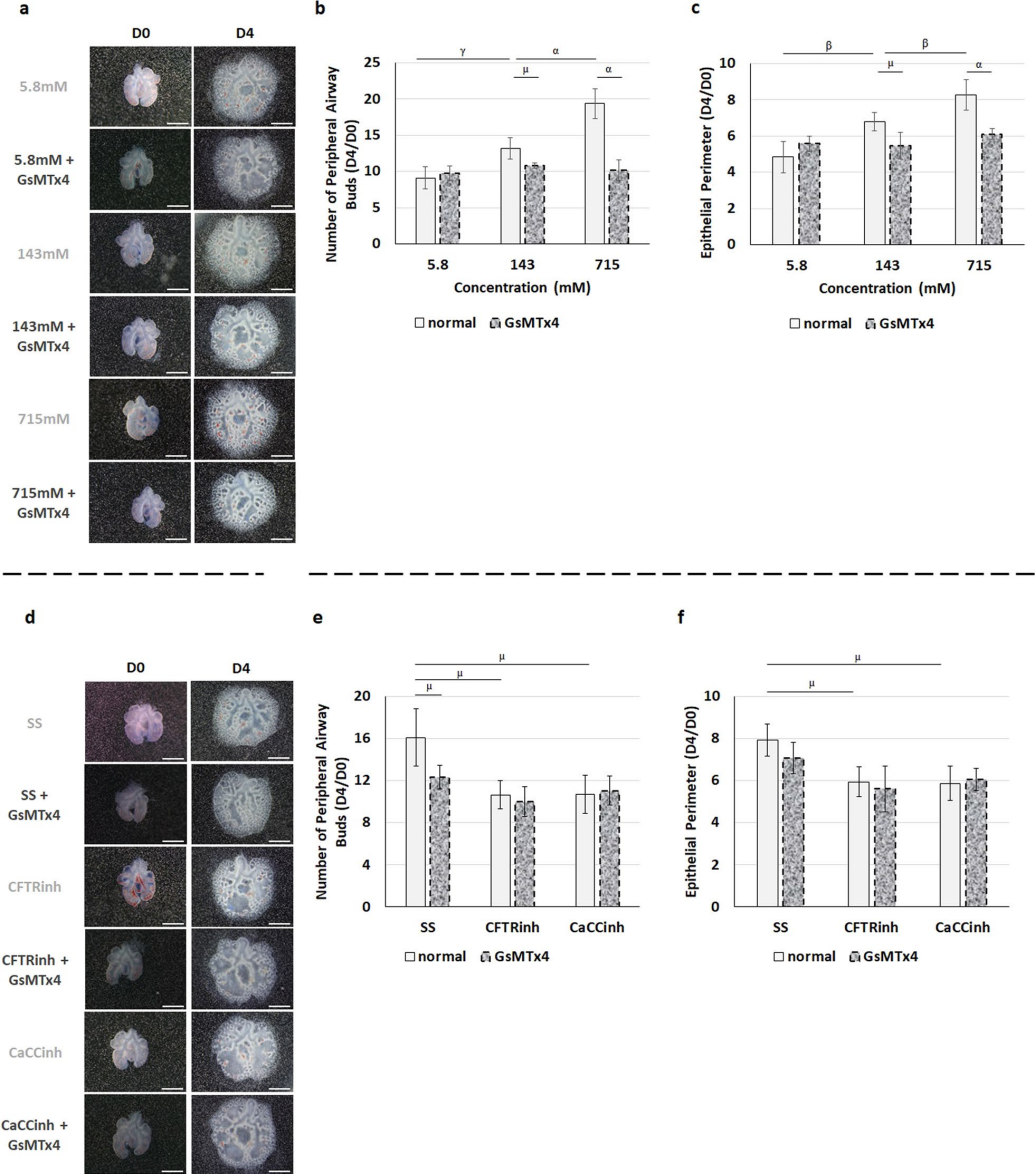
PIEZO1 and PIEZO2 mediate the effects of manipulating intraluminal chloride. **a**–**c** Upper panel represents the main cumulative effect of intraluminal injection of distinct chloride concentrations ([Cl^−^]) 5.8, 143, or 715 mM Cl^−^ and the medium supplementation with GsMTx4 at day0 (D0) and day2 (D2). **a** Represents lung explants at D0 and D4 for the different [Cl^−^]. **b**, **c** Morphometric analysis of **b** peripheral airway buds and **c** epithelial perimeter. **d**–**f** Lower panel shows the additional effect of PIEZO1/2 inhibition after intraluminal injection of Cl^−^ channels inhibitors: cystic fibrosis transmembrane conductance regulator inhibitor172 (CFTRinh) to CFTR; and calcium-dependent Cl^−^ channel inhibitor A01 (CaCCinh) to CaCCs. **d** represents the fetal lung explants at D0 and D4 for the distinct Cl^−^ channels inhibitors. **e**, **f** Morphometric analysis of **e** peripheral airway buds and **f** epithelial perimeter. 143 mM Cl^−^ and standard solution (SS) identified the control condition for [Cl^−^] and Cl^−^ channels inhibitors, respectively. White and dotted rectangles represent the medium supplementation with and without GsMTx4, respectively. n ≥ 4 were used per antibody/condition. Results are expressed as the ratio of D4 and D0 (D4/D0) and presented as mean ± SD. Symbols indicate the main effects and non-redundant interactions of the two-way ANOVA. p < ^α^0.0001, ^β^0.001, ^γ^0.01, ^µ^0.05

The morphologic analysis showed a similar number of peripheral airway buds and epithelial perimeter after GsMTx4 medium supplementation and intraluminal injection of 5.8, 143, or 715 mM Cl^−^ (Fig. 4a–c). Indeed, the GsMTx4 inhibits branching morphogenesis at 143 and 715 mM Cl^−^, whereas similar branching morphogenesis was observed at 5.8 mM Cl^−^ in normal and supplemented lungs. No significant differences in lung growth were detected between 5.8 mM Cl^−^ without GsMTx4 and 5.8, 143, or 715 mM with GsMTx4 (Fig. 4a–c). Concerning the Cl^−^ channels inhibition, the synchronous SS injection and PIEZO1/PIEZO2 downregulation triggered a relevant decrease in the number of peripheral airway buds (Fig. 4d, e). Oppositely, the decrease in branching morphogenesis induced by CFTR or CaCCs inhibitors (compared to SS normal) was unchanged by PIEZO1/PIEZO2 inhibition (Fig. 4d–f).

**Fig. 5:**
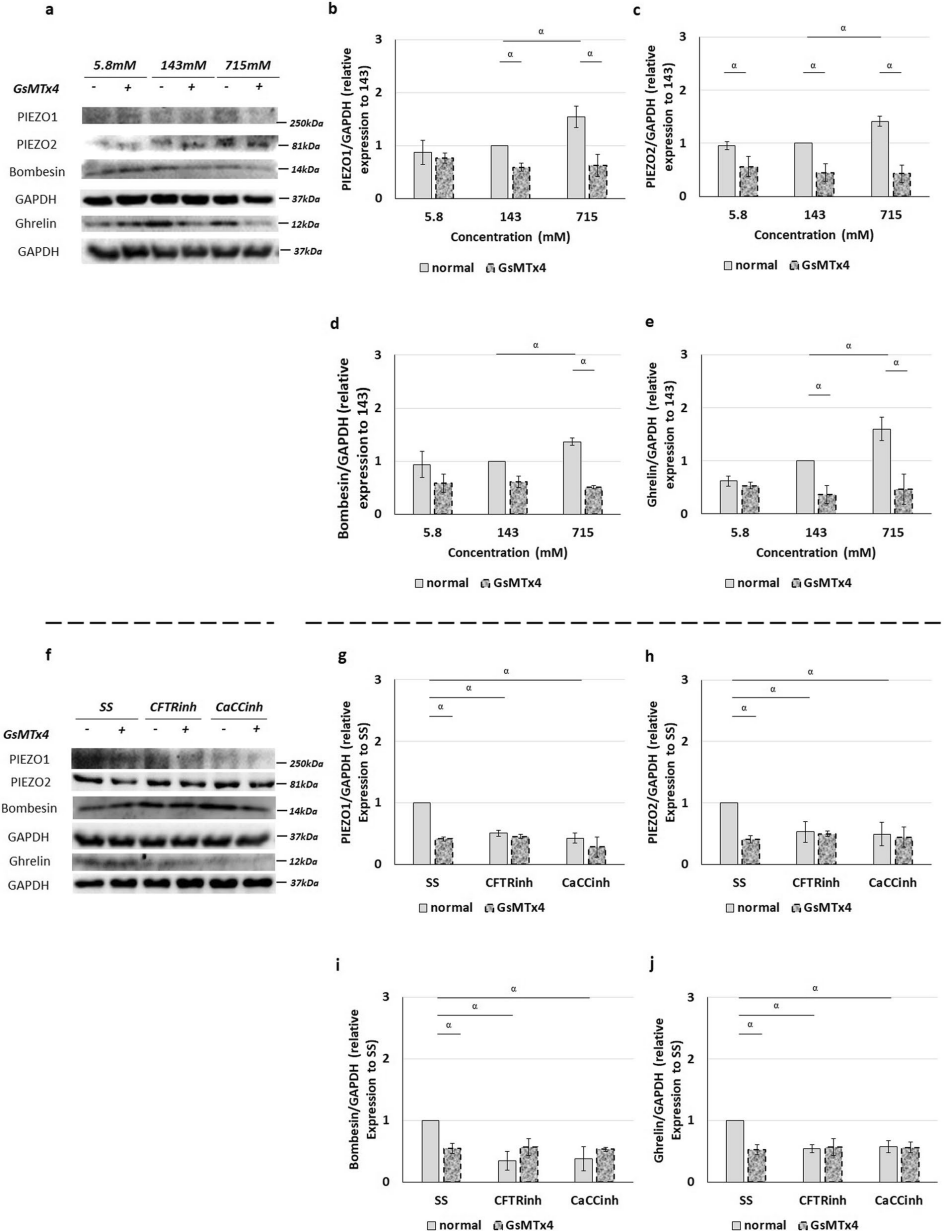
Decrease of PIEZO1 and PIEZO2 expression removes the molecular dynamics triggered by intraluminal chloride concentration. **a**–**e** Upper panel represents the main cumulative effect of intraluminal chloride concentration ([Cl^−^]) and medium supplementation with (dotted rectangles) and without (white rectangles) GsMTx4. **a** Examples of representative blots are shown. **b**–**e** Protein expression levels for **b** PIEZO1, **c** PIEZO2, **d** bombesin, and **e** ghrelin are indicated. **f**–**j** Lower panel shows the additional effect of GsMTx4 after intraluminal injection of Cl^−^ channels inhibitors: cystic fibrosis transmembrane conductance regulator inhibitor172 (CFTRinh) to CFTR; and calcium-dependent Cl^−^ channel inhibitor A01 (CaCCinh) to CaCCs. **f** Examples of representative blots are shown. **g**–**j** Relative expression levels of **g** PIEZO1, **h** PIEZO2, **i** bombesin, and **j** ghrelin. 143 mM Cl^−^ and standard solution (SS) represent the control condition for [Cl^−^] and Cl^−^ channels inhibitors, respectively. n ≥ 4 were used per antibody/condition. Results are presented as mean ± SD. Symbols indicate the main effects and non-redundant interactions of the two-way ANOVA. p < ^α^0.0001

To better identify the PIEZO1/2 function in fetal lung growth, the relative expression levels of PIEZO1, PIEZO2, ghrelin, and bombesin were also evaluated at the abovementioned conditions. GsMTx4 treated-lung presented a decrease in PIEZO1 (Fig. 5a, b), PIEZO2 (Fig. 5c), bombesin (Fig. 5d), and ghrelin (Fig. 5e) expression at 143 and 715 mM Cl^−^ when compared with the GsMTx4 untreated-lungs (Fig. 5a–e). In contrast, PIEZO1 (Fig. 5a, b), PIEZO2 (Fig. 5c), bombesin (Fig. 5d), and ghrelin (Fig. 5e) expression levels remain unaffected comparing the injection of 5.8 mM Cl^−^ with and without GsMTx4.

Concerning the Cl^−^ channels inhibitors, we reported the GsMTx4 as an inhibitor of PIEZO1 (Fig. 5f, g), PIEZO2 (Fig. 5h), bombesin (Fig. 5i), and ghrelin (Fig. 5j) expression after intraluminal injection of SS. On the other hand, the simultaneous inhibition of PIEZO1/PIEZO2 and CFTR or CaCC injection had no additional effect on the relative expression levels of PIEZO1 (Fig. 5f, g), PIEZO2 (Fig. 5h), bombesin (Fig. 5i) or ghrelin (Fig. 5j).

### The molecular effect of intraluminal injections in airway smooth muscle cells

**Fig. 6:**
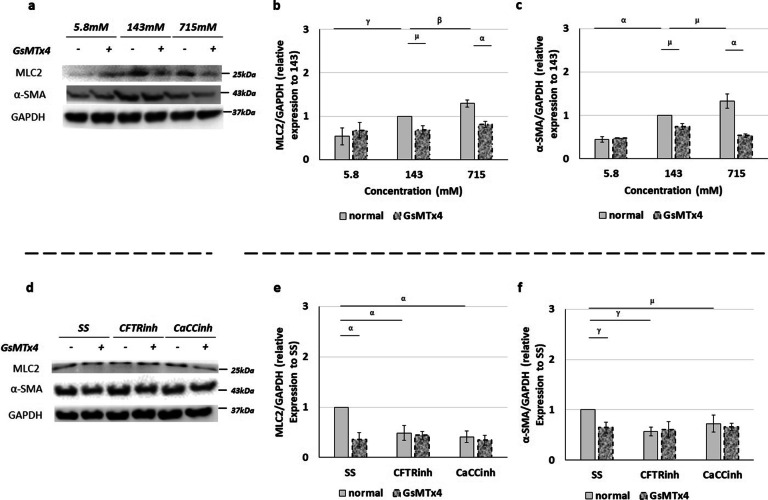
Molecular effect of intraluminal chloride and GsMTx4 in smooth muscle cells. **a-c** Upper panel represents the main cumulative effect of intraluminal chloride concentration ([Cl^−^]) and medium supplementation with and without GsMTx4. **a** Examples of representative blots are shown. **b**, **c** Protein expression levels for **b** myosin light chain 2 (MLC2), and **c** alpha-smooth muscle actin (α-SMA) are quantified. **d–f** Lower panel shows the additional effect of PIEZO1/2 inhibition after intraluminal injection of Cl^−^ channels inhibitors: cystic fibrosis transmembrane conductance regulator inhibitor172 (CFTRinh) to CFTR and; calcium-dependent Cl^−^ channel inhibitor A01 (CaCCinh) to CaCCs. **d** Examples of representative blots are shown. **e–f** Relative expression levels of **e** MLC2, and **f** α-SMA are displayed. 143 mM Cl^−^ and standard solution (SS) represent the control condition for [Cl^−^] and Cl^−^ channels inhibitors, respectively. White and dotted rectangles represent the medium supplementation with and without GsMTx4, respectively. Each lane represents a pooled-tissue sample, and the relative expression levels were determined against GAPDH. n ≥ 4 were used per antibody/condition. Results are presented as mean ± SD. Symbols indicate the main effects and non-redundant interactions of the two-way ANOVA. p < ^α^0.0001, ^β^0.001, ^γ^0.01, ^µ^0.05

Previous work reported a morphological interaction between branching morphogenesis and peristaltic airway contractions. Thus, we then assessed the molecular profile of MLC2 and α-SMA at the above-mentioned experimental conditions (Fig. 6a–c). Our findings revealed an opposite effect in the expression of MLC2 (Fig. 6b) and α-SMA (Fig. 6c) after injection of 5.8 or 715 mM Cl^−^. Indeed, when compared with 143 mM Cl^−^, 5.8 mM inhibits whereas 715 mM Cl^−^ promotes MLC2 (Fig. 6b) and α-SMA expression (Fig. 6c). Finally, the depletion of PIEZO1/PIEZO2 expression achieved by GsMTx4 medium supplementation displayed a similar decrease in the relative expression levels of MLC2 (Fig. 6b) and α-SMA (Fig. 6c) at 143 and 715 mM Cl^−^ observed at D4. No differences in the relative expression levels were detected after injection of 5.8 mM Cl^−^ with and without GsMTx4.

Regarding the Cl^−^ channel inhibitors, after the intraluminal injection of CFTRinh or CaCCinh versus SS, a significant inhibitory effect was uncovered in MLC2 (Fig. 6d, e) and α-SMA (Fig. 6f). In addition, GsMTx4 medium supplementation only decreased the relative expression levels of MLC2 (Fig. 6e) and α-SMA (Fig. 6f) previously observed after SS intraluminal. No additional effect in MLC2 (Fig. 6e) or α-SMA (Fig. 6f) were visualized after simultaneously GsMTX4 medium supplementation and intraluminal injections of CFTRinh or CaCCinh (Fig. 6d–f).

## Discussion

The fetal lung develops as a fluid-filled organ that maintains the lung in a constantly distended state, stimulating its growth and maturation [60–63]. Unfortunately, the difficulty of capturing or recapitulating the in vivo morphological dynamics in the lab hinders the study of the underlying mechanisms, particularly at early developmental stages. In this context, ex vivo lung explant cultures are an asset since it maintains the in vivo physiologic architecture and the cellular interactions observed at the pseudoglandular or branching stage [57, 64]. Bearing this in mind, we established effective intraluminal injections at D0 and D2 and observed dynamic movements in the lumen at D4, indicating the existence of lung liquid in the ex vivo model and validating this approach as a valuable method for studying lung fluid composition in branching morphogenesis.

**Fig. 7:**
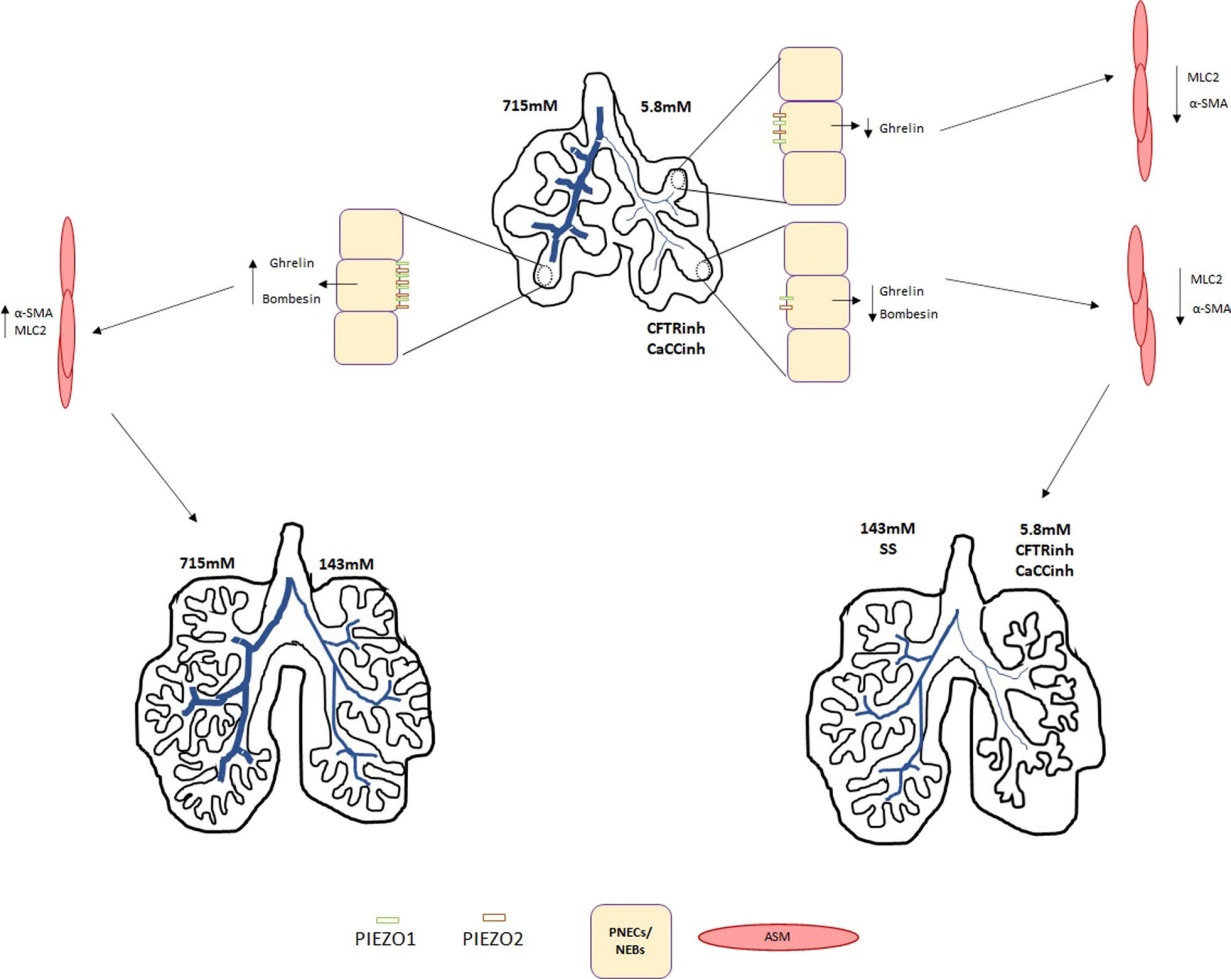
Schematic representation of the role of intraluminal chloride concentrations in fetal branching morphogenesis. Increasing concentrations of intraluminal [Cl^−^] leads to increased branching morphogenesis and expression of neuroendocrine products, PIEZO channels, and smooth muscle markers. Pharmacological inhibition experiments show that these effects require PIEZO and Cl^−^ channel activation. α-SMA, alpha-smooth muscle actin; CaCCinh calcium-dependent Cl^−^ channel inhibitorA01; CFTRinh, cystic fibrosis transmembrane conductance regulator inhibitor172; MLC2, myosin light chain 2; SS, standard solution

On the strongly evidenced premise that the Cl^−^ movement in the epithelium is an inductor of Na^+^ and water movements in the same direction [22–25, 65–67], we manipulated the intraluminal lung fluid composition by injecting different [Cl^−^] or Cl^−^ channel inhibitors and then analyzing the branching morphogenesis at D4. Our findings demonstrated that intraluminal [Cl^−^] was able to regulate fetal lung growth. In fact, the increase of luminal [Cl^−^] stimulated branching morphogenesis, whereas a significant decrease was observed after depletion of [Cl^−^] at 5.8 mM Cl^−^ (Fig. 7). No significant effect was identified in branching morphogenesis at 29 mM Cl^−^. These results further indicate distinct capacities for the injected solutions to imbalance the extracellular versus intracellular ionic charges, as previously predicted by mathematical models. For instance, the Gibbs-Donnan effect establishes that charged particles near a semi-permeable membrane sometimes fail to distribute evenly across the two sides of the membrane. In addition, the potential equilibrium for Cl^−^ calculated from the Nernst equation [68] supports our knowledge regarding the differences between the intracellular versus extracellular manipulation of [Cl^−^]. Both effects were demonstrated in neuronal membranes and point to features governing fluid exchange between the intra and extracellular environment of cells [69, 70]. In our model, the manipulation of extracellular [Cl^−^] suggests a range, higher than 29 mM Cl^−^, compatible with normal fetal lung development, indicating an interesting effect for the in vivo lung fluid production and maintenance of a suitable environment for lung cells. Curiously, experiments in fetal sheep have demonstrated a direct link between reduced distension due to fluid loss and lung hypoplasia. Conversely, tracheal obstruction in utero leads to fluid accumulation and more rapid lung growth [71]. These differences in pressure between the airway lumen and surrounding tissue are essential for normal airway development, with tension and mechanical stretch playing additional roles in cellular differentiation and airway growth [72].

Regarding the Cl^−^ channel inhibitors, fluid secretion into the airway lumen is driven by Cl^−^ transport, which is mediated by at least two types of anion channels, CaCC and CFTR, that respond to different stimuli: intracellular Ca^2+^ and cAMP, respectively [73–75]. Conversely, an interdependent function for CFTR and TMEM16A has been demonstrated in adult mice and human respiratory and intestinal epithelial cells [76]. Our study suggests a comparable strong effect of both CFTR and CaCC in lung fluid production and branching morphogenesis. In contrast, the reported unchanged branching morphogenesis after A9C injection, an inhibitor of TMEM16A [77], indicates an insufficient or quickly compensated function for TMEM16A in lung fluid secretion. Brennan et al. [20] also described TMEM16A and bestrophin1 as insufficient for Ca^2+^-stimulated lung fluid secretion in the mouse fetal lung. Finally, the molecular inhibition of ionic channels, such as CFTR, ClC2, or CaR, critical for lung fluid secretion, induces key defects in vivo and ex vivo branching morphogenesis [18, 20, 21, 26, 27, 30, 78] that reinforce the value of our ex vivo model for the study of intraluminal fluid in fetal lung growth.

Collectively, these findings prompt us to go further in the inherent molecular/cellular mechanisms. As such, our data demonstrated that PIEZO1 and PIEZO2 colocalize with PGP9.5, a molecular marker for PNECs/NEBs. Interestingly, PIEZO1 and PIEZO2 receptors are sensors for mechanical stretch, like pressure, with major roles in regulating blood pressure and respiratory function at birth, respectively [41, 45, 79]. In contrast, PNECs/NEBs are described as airway sensors with poorly understood functions [40]. Literature also shows that the secreted neuroendocrine products, ghrelin, and bombesin, are promotors of the in vivo and ex vivo fetal lung growth [35–39]. To further investigate these dynamics, neuroendocrine products (ghrelin, bombesin) and mechanoreceptors (PIEZO1 and PIEZO2) were quantified at the abovementioned conditions. We found that the intraluminal [Cl^−^] modulates ghrelin, bombesin, PIEZO1, and PIEZO2 expression in branching morphogenesis. Briefly, the decrease of luminal [Cl^−^], 5.8 mM Cl^−^, was an inhibitor of ghrelin with no significant effects on the expression of the remaining markers (Fig. 7). Surprisingly, 715 mM Cl^−^ stimulated ghrelin, bombesin, PIEZO1, and PIEZO2 expression, whereas the luminal injection of CFTRinh or CaCCinh inhibited fetal lung growth and equally inhibited the four markers (Fig. 7).

Dickson et al. [9] described the lung liquid as a regulator of fetal lung growth in a mechanism independent of the lung fluid secretion and suggested that the lung is unable to respond to alterations in lung liquid volume. Indeed, the decrease in pulmonary growth was related to reduced tracheal pressure and tracheal efflux rate in the fetal sheep model. Now, our analysis revealed that PIEZO1, PIEZO2, and bombesin were overexpressed at 715 mM Cl^−^ and unchanged at 5.8 mM Cl^−^, when compared with the basal [Cl^−^], 143 mM. As such, it is important to define the fundamental responses in fetal lung growth after PIEZO1/PIEZO2 inhibition. For that, we inhibited the PIEZO1/PIEZO2 on the day of intraluminal injections by GsMTx4. We demonstrated a significant inhibition of neuroendocrine products and branching morphogenesis after GsMTx4 medium supplementation. More importantly, this outcome was independent of the intraluminal [Cl^−^]. In fact, apart from 5.8 mM Cl^−^ where no significant differences between GsMTx4-treated and -untreated lungs were observed, the intraluminal injection of 143, 715 mM Cl^−^ or SS with the synchronized inhibition of PIEZO1/PIEZO2 by GsMTx4 similarly decreased the morphological and molecular profiles. These findings indicate that PIEZO1 and PIEZO2 are major regulators of the mechanosensory pathway in branching morphogenesis. A recent publication showed PIEZO2 as a regulator of pulmonary function in neonates and adults [41]. Indeed, the global and sensory neuron-specific ablation of mechanically activated ion channel PIEZO2 causes respiratory distress and death in newborn mice. In contrast, the induced ablation of PIEZO2 in sensory neurons of adult mice causes decreased neuronal responses to lung inflation and an impaired Hering-Breuer mechanoreflex [41]. In this context, our investigation suggests that PIEZO1/PIEZO2, present in PNECs/NEBs, act as sensors of branching morphogenesis in fetal lung development.

Physiology has shown that the movement of intraluminal fluid through epithelial tubules is a consequence of the peristaltic activity of fetal airway smooth muscle (ASM) that maintains positive pressure in the lumen area to keep the tubules in a distended state [62]. The formation of new airspaces during branching morphogenesis early in gestation is closely followed by the differentiation of mesenchymal cells into ASM cells. Evidenced by cellular expression of the contractile protein α-SMA as an early differentiation marker [80], ASM progenitor cells have been identified in both the proximal and distal lung mesenchyme [81, 82]. This differentiation of ASM simultaneously produces the MLC filaments in fetal lungs [31–34, 83–85]. To determine the molecular effect of intraluminal Cl^−^ composition in airway smooth muscle cells, we evaluated α-SMA and MLC2 at the aforementioned experimental condition with and without GsMTx4. We observed that the increase of [Cl^−^] and branching morphogenesis relates to the overexpression of MLC2 and α-SMA. Conversely, the decrease in α-SMA and MLC2 were associated with reduced [Cl^−^] and branching morphogenesis (Fig. 7). One can argue that the increase/decrease in ASM markers may either alter the frequency or the force of peristaltic airway contractions thus altering peristalsis effect on lung growth [86].

Overall, these results indicated that the intraluminal composition and the neuroendocrine activation act upstream of airway smooth muscle contraction and branching morphogenesis. Interestingly, the hypoplastic phenotype observed in the CDH context was connected to a decrease in α-SMA and MLC2 from the pseudoglandular-to-canalicular stage [32]. In contrast, tracheal occlusion in in vivo mouse model was an inductor of α-SMA and MLC2 expression at the later canalicular stage [87], suggesting that the PIEZO1/PIEZO2 pathway may be a potential target for the treatment of fetal pulmonary hypoplasia.

## Conclusions

Our findings offer a mechanistic basis for previous in vivo data that reported an excess of fluid drainage during fetal life or a decreased fluid pressure associated with lung hypoplasia with underbranched lungs. Here, we describe key information on the specific pathway by which the intraluminal Cl^−^ composition regulates fetal lung growth. We demonstrate that the intraluminal [Cl^−^] activates PNECs/NEBs through PIEZO1/PIEZO2 mechanoreceptors that, in turn, regulate the expression of ghrelin, bombesin, α-SMA, and MLC2 thus regulating fetal lung growth (Fig. 7).

## Supplementary Information

**Additional file 1: Table S1.** Summary of chemical compounds in injected solution used for manipulation of the intraluminal fluid. **a** show the chemical concentration by compound for standard solution (SS) and crescent chloride concentrations, [Cl^−^]: 5.8, 29, 143, and 715 mM. **b** demonstrates the ionic composition in terms of chloride (Cl^−^), potassium (K^+^), magnesium (Mg^2+^) and calcium (Ca^2+^) in SS, 5.8, 29, 143 and 715 mM Cl^−^.

**Additional file 2: Movie S1.** Injection of standard solution into the lumen of ex vivo lung explant cultures at day0 (D0).

**Additional file 3: Movie S2.** Injection of standard solution into the lumen of ex vivo lung explant cultures at day2 (D2).

**Additional file 4: Movie S3.** Dynamic intraluminal movement in ex vivo lung explant cultures at day4 (D4).

## Data Availability

The authors declare that the data supporting the findings of the present study are available within the manuscript or from the corresponding author upon reasonable request.

## Abbreviations

[Cl^−^]: Chloride concentration
A9C: Anthracene-9-carboxylic acid
BSA: Bovine serum albumin
Ca^2^^+^: Calcium
CaCC: Calcium-dependent chloride channel
CaCCinh: Calcium-dependent chloride inhibitorA01
CaCl_2_: Calcium chloride
CaR: Calcium receptor
CDH: Congenital diaphragmatic hernia
CFTR: Cystic fibrosis transmembrane conductance regulator
CFTRinh: Cystic fibrosis transmembrane conductance regulator inhibitor172
Cl^−^: Chloride
ClC_2_: Chloride channel2
CO_2_: Carbon dioxide
D0: Day 0;
DAPI: 4′,6-Diamidino-2-phenylindole
DMEM: Dulbecco's Modified Eagle Medium
DMSO: Dimethyl sulfoxide
E: Embryonic day
FBS: Fetal bovine serum
FETO: Fetoscopic endoluminal tracheal occlusion
GAPDH: Glyceraldehyde-3-phosphate dehydrogenase
HEPES: 4-(2-Hydroxyethyl)-1-piperazineethanesulfonic acid
K^+^: Potassium
KCl: Potassium chloride
Mg^2^^+^: Magnesium
MgCl_2_: Magnesium chloride
MgSO_4_: Magnesium sulfate
MLC_2_: Myosin light chain 2
Na/K/2Cl: Sodium–potassium-2chloride cotransporter
Na/K-ATPase: Sodium–potassium adenosine triphosphatase pump
NaCl: Sodium chloride
NEBs: Neuroepithelial bodies
PBS: Phosphate buffered saline
PGP9.5: Protein gene product 9.5
PNECs: Pulmonary neuroendocrine cells
RT: Room temperature
SS: Standard solution
TMEM16A: Transmembrane protein 16A
α-SMA: Alpha-smooth muscle actin
