# Supplementary figures and images for: Intraluminal chloride regulates lung branching morphogenesis: involvement of PIEZO1/PIEZO2
Source: Respir Res. 2023 Feb 5;24:42. doi: 10.1186/s12931-023-02328-2 (PMC9901166; doi:10.1186/s12931-023-02328-2)

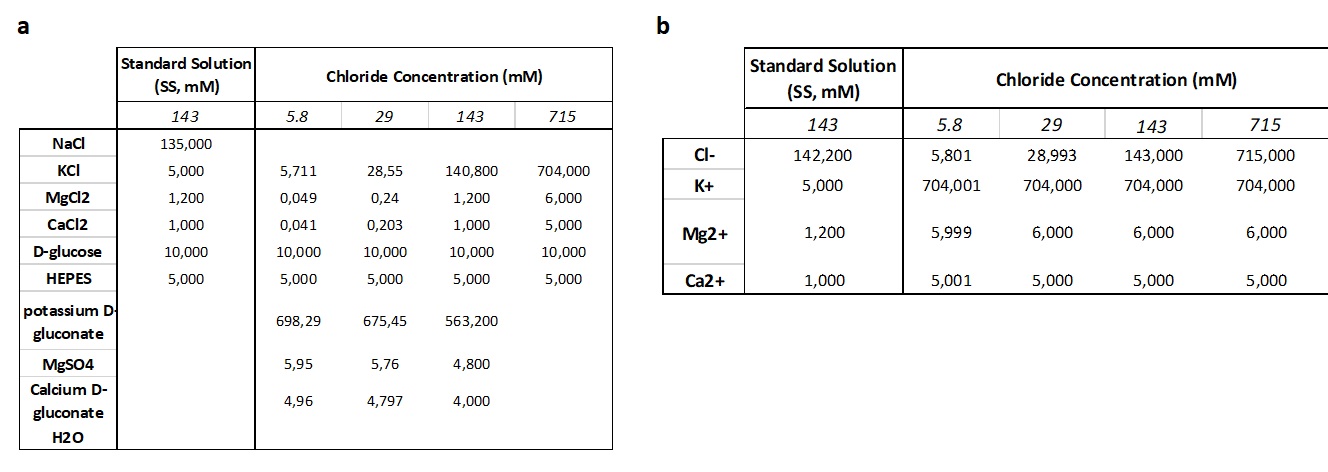

Supplement: Supplementary file 1 — Additional file 1: Table S1. Summary of chemical compounds in injected solution used for manipulation of the intraluminal fluid. a show the chemical concentration by compound for standard solution (SS) and crescent chloride concentrations, [Cl−]: 5.8, 29, 143, and 715 mM. b demonstrates the ionic composition in terms of chloride (Cl−), potassium (K+), magnesium (Mg2+) and calcium (Ca2+) in SS, 5.8, 29, 143 and 715 mM Cl−. [file 12931_2023_2328_MOESM1_ESM.jpg]
